# The prevalence of *Chlamydia trachomatis* infection in Australia: a systematic review and meta-analysis

**DOI:** 10.1186/1471-2334-12-113

**Published:** 2012-05-14

**Authors:** Dyani Lewis, Danielle C Newton, Rebecca J Guy, Hammad Ali, Marcus Y Chen, Christopher K Fairley, Jane S Hocking

**Affiliations:** 1 School of Population Health, University of Melbourne, Carlton, Victoria, Australia; 2 The Kirby Institute, University of New South Wales, Darlinghurst, New South Wales, Australia; 3 Melbourne Sexual Health Centre, Alfred Hospital, Carlton, Victoria, Australia

**Keywords:** Chlamydia, Meta-analysis, Prevalence, Systematic review

## Abstract

**Background:**

*Chlamydia trachomatis* is a common sexually transmitted infection in Australia. This report aims to measure the burden of chlamydia infection by systematically reviewing reports on prevalence in Australian populations.

**Methods:**

Electronic databases and conference websites were searched from 1997–2011 using the terms ‘*Chlamydia trachomatis*’ OR ‘chlamydia’ AND ‘prevalence’ OR ‘epidemiology’ AND ‘Australia’. Reference lists were checked and researchers contacted for additional literature. Studies were categorised by setting and participants, and meta-analysis conducted to determine pooled prevalence estimates for each category.

**Results:**

Seventy-six studies met the inclusion criteria for the review. There was a high level of heterogeneity between studies; however, there was a trend towards higher chlamydia prevalence in younger populations, Indigenous Australians, and those attending sexual health centres. In community or general practice settings, pooled prevalence for women <25 years in studies conducted post-2005 was 5.0% (95% CI: 3.1, 6.9; five studies), and for men <30 years over the entire review period was 3.9% (95% CI: 2.7, 5.1; six studies). For young Australians aged <25 years attending sexual health, family planning or youth clinics, estimated prevalence was 6.2% (95% CI: 5.1, 7.4; 10 studies) for women and 10.2% (95% CI: 9.5, 10.9; five studies) for men. Other key findings include pooled prevalence estimates of 22.1% (95% CI: 19.0, 25.3; three studies) for Indigenous women <25 years, 14.6% (95% CI: 11.5, 17.8; three studies) for Indigenous men <25 years, and 5.6% (95% CI: 4.8, 6.3; 11 studies) for rectal infection in men who have sex with men. Several studies failed to report basic demographic details such as sex and age, and were therefore excluded from the analysis.

**Conclusions:**

*Chlamydia trachomatis* infections are a significant health burden in Australia; however, accurate estimation of chlamydia prevalence in Australian sub-populations is limited by heterogeneity within surveyed populations, and variations in sampling methodologies and data reporting. There is a need for more large, population-based studies and prospective cohort studies to compliment mandatory notification data.

## Background

*Chlamydia trachomatis* (here after referred to as chlamydia) is the most commonly diagnosed bacterial sexually transmitted infection (STI) in Australia [1,2]. In women, chlamydia can lead to serious and costly health consequences, particularly if the infection ascends from the endocervix to the upper genital tract and causes pelvic inflammatory disease (PID), which may result in fallopian tube scarring, ectopic pregnancy, tubal infertility and chronic pelvic pain [3-6]. Chlamydia also causes epididymo-orchitis in men [7,8] and can act as a co-factor in increasing the risk of HIV transmission in both men and women [9].

Genital chlamydial infection became a notifiable disease in 1991 in all Australian States and Territories except for NSW, which introduced mandatory notification in 1997 [1]. Notification rates have been steadily rising over the past decade [2], with rates highest among young people. Chlamydia notification rates are highest in the Northern Territory [2], which records high rates among Indigenous Australians [10]. Given that chlamydia is asymptomatic in up to 90% of infections, testing rates remain low (less than 10% in the younger age groups) [11], and re-infections are common, notification data greatly underestimate the true burden of infection. It is therefore important to gather high quality, region-specific epidemiological data to estimate the prevalence of chlamydia in Australian populations. A review published in 2005 reported an overall prevalence of chlamydia of 4.6% (95%CI: 4.4%, 4.8%) [12]; however, there has been considerable further chlamydia epidemiological research conducted in Australia since then. This review examines the available data on the prevalence of chlamydia across Australia and provides an up-to-date picture of the burden of chlamydia in Australian communities. This information will help to inform future clinical practice and screening policies.

## Methods

### Review strategy

The electronic bibliographic database Medline was searched for English-language articles published between 1997 and July 2011. Reference lists of selected studies were also checked for other potentially relevant studies. Proceedings of the Australian Sexual Health Conference were also reviewed to identify potential unpublished studies. The PRISMA statement for preferred reporting of systematic reviews and meta-analyses was used as a guide to conducting the review and analysis [13].

The following search terms were used: (*Chlamydia trachomatis* OR chlamydia) AND (prevalence OR epidemiology) AND Australia.

The studies were reviewed and information extracted by two authors independently; disagreements were resolved by discussion and consensus. Criteria for inclusion were:

· Individuals were tested during the review period, 1997 to July 2011;

· The study presented the number of infections and the total number of individuals tested;

· Nucleic acid amplification tests (NAAT) were used.

Studies were excluded for the following reasons:

· The study described self-reported STI diagnoses;

· Tests other the NAAT were used [14];

· The type of test was not specified and testing was performed prior to 2000.

Studies conducted partially within the review period were included if year-by-year data was presented, or if the time was substantially within the review period and NAAT testing was used throughout.

Variables extracted from each study included geographical location, year of the study, setting, participants, specimen type, gender, number of participants and number testing positive. Where possible, the age (mean or range), participation rates, and age- and sex-specific prevalence data were also extracted. For studies that did not report them, 95% confidence intervals (CI) were calculated using exact methods in STATA 11 (StataCorp, College Station, TX, USA).

Results were classified by setting and participants, with studies grouped into the following categories: general practice (GP) or community-based populations; clients of sexual health, and family planning centres, youth centres, and other medical clinics; pregnant women; Indigenous Australians; men who have sex with men (MSM); and high-risk populations. Chlamydia positivity (number testing positive divided by total number tested) was used as a surrogate measure of chlamydia prevalence and on this basis, the term ‘prevalence’ will be used throughout this review.

### Meta-analysis

Where appropriate and where data was available, female and male data were pooled separately for meta-analysis (STATA 11; StataCorp, College Station, TX, USA). Studies reporting combined prevalence data for male and female participants were excluded from meta-analysis. The I^2^ test was used to estimate the proportion of total variability in point estimates attributed to heterogeneity other than that due to chance. Data were pooled according to the level of between-study heterogeneity, using the following strategy [15]:

· I^2^ < 25%, fixed effects meta-analysis to estimate the common prevalence (95% CI), assuming that all or most between-study variability is due to chance;

· I^2^ 25–75%, random effects meta-analysis to estimate the average prevalence (95% CI);

· I^2^ > 75%, heterogeneity too great for summary estimate to be calculated.

Possible reasons for heterogeneity were explored by stratifying results by study setting.

## Results

**Figure 1:**
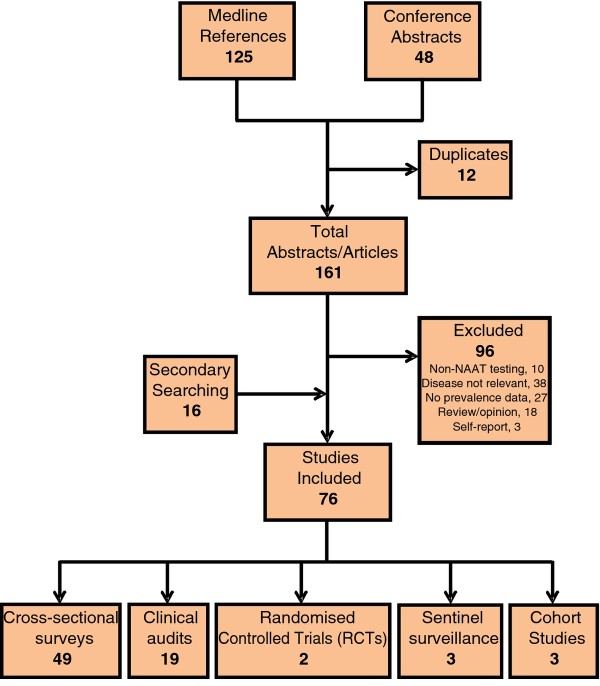
**Systematic review of chlamydia prevalence in Australian populations.** Flow chart of inclusions and exclusions from the systematic literature review.

A total of 76 studies, described in 78 published articles and six conference abstracts, from 129 articles and 48 abstracts fit the inclusion criteria (Figure 1; Table 1; Additional files 1, 2, 3, 4, and 5). The majority of studies (49 of 76) estimated chlamydia prevalence through cross-sectional surveys, with sample sizes of between 44 and 2817 participants (median 346). There were also 19 clinical audits (median sample size 505; range 80–26,097), three cohort studies (median 457; range 456–1642), three sentinel surveillance reports (median 30,516; range 3551–69,927), and two randomised controlled trials (RCTs; mean 1218; range 843–1593).

**Table 1:** Studies reporting chlamydia prevalence data, identified in general practice or community settings

**Study**	**Location**	**Participants**	**Study design**	**Specimen type**	**Response rate (%)**	**Sex**	**Age (years)**	**Study period**	**Tested (n)**	**Positive (n)**	**Prevalence % (95% CI)**
**General Population**											
Hocking [16]	VIC (Melb)	Women recruited from the telephone directory	Cross-sectional survey	Urine	43	F	18–35	2003–2004	657	6	0.9 (0.3, 2.0)
							(18–24)		135	5	3.7 (1.2, 8.4)
							(25–35)		489	1	0.2 (0.0, 1.1)
**Community**											
Debattista [17]	QLD (Bris)	University students at residential colleges	Cross-sectional survey	Urine	30–50	F	<25	1997	178	2	1.1 (0.1, 4.0)*
						M			96	0	0 (0.0, 3.8)*
Debattista [18]	QLD (Bris)	High school students	Cross-sectional survey	Urine	30–50	F	15–18	1998–2001	516	12	2.3 (1.2, 4.0)**
						M		(1998)	170	2	1.2 (0.2, 4.6)*
								(1999)	147	5	3.4 (1.3, 8.2)*
								(2000)	101	0	0 (0.0, 4.6)*
								(2001)	98	5	5.1 (1.9, 12.1)*
								1998–2001	658	3	0.5 (0.1, 1.3)**
								(1998)	339	0	0 (0.0, 1.4)*
								(1999)	132	1	0.8 (0.0, 4.8)*
								(2000)	94	0	0 (0.0, 4.9)*
								(2001)	93	2	2.2 (0.4, 8.3)*
Bowden [19]	ACT	High school students	Cross-sectional survey	F: urine/ swab	31	F	15–20	2002–2003	273	2	0.7 (0.1, 2.6)
						M			179	3	1.7 (0.3, 4.8)
				M: urine							
Davis [20]	ACT	Tertiary students recruited at outreach screening services	Clinical outreach pilot	Urine	29.4	M/F	16–26	2007	445	8	1.8 (0.1, 3.5)*
Gold [21]	VIC (rural/ regional)	Men attending rural football clubs	Cross-sectional survey	Urine	85	M	16–29	2006–2007	77	3	3.9 (0.8, 11.0)
Wade [22]	VIC (Melb)	Heterosexual men attending sporting clubs	Cross-sectional survey	Urine	87	M	16–29	n.r.	47	4	8.5 (2.4, 20.4)**
Buhrer-Skinner [23]	QLD (Towns-ville)	Individuals attending outreach screening services set up in a variety of community locations	Cross-sectional survey	M: urine	n.r.	M/F	21^A^	2004–2005	303	15	5.0 (2.8, 8.0)*
				F: urine/ swab		(M)	25		75	5	Army: 6.7 (2.2, 14.9)
						(M/F)	21		95	5	University: 5.3 (1.7, 11.9)
											
						(M/F)	17		68	0	High school festivities: 0 (0, 5.3)
						(M/F)	25		65	5	Backpackers: 7.7 (2.5, 17.0)
Kong [24]; Kong [25]	VIC (rural/ regional)	Young people attending sporting clubs	Cross-sectional survey	Urine	>95	F	16–29	2007	121	9	7.4 (3.5, 13.7)**
						M			426	19	4.5 (2.7, 6.9)
Sacks-Davis [26]	VIC (Melb)	Young people attending a music festival	Cross-sectional survey	Urine	21	F	16–29	2009	46	0	0 (0.0, 7.7)*
						M			21	1	4.8 (0.1, 23.8)*
Davies [27]	NSW (Sydney)	Young international backpackers recruited at hostels	Cross-sectional survey	F: swab	45.7	F	18–30	2009	207	8	3.9 (1.7, 7.5)
				M: urine	50.2	M	(≤25)		166	5	3.0 (1.0, 6.9)*
							(26–30)		41	3	7.3 (1.5, 19.9)*
							18–30		225	7	3.1 (1.3, 6.3)
							(≤25)		164	6	3.7 (1.4, 7.8)*
							(26–30)		61	1	1.6 (0.4, 8.8)*
**General Practice**											
Heal [28]	QLD (Mackay)	Young people attending general practices	Cross-sectional survey	Urine	68	F/M	18–24	2001	443	17	3.8 (2.3, 6.1)*
Chiang [29]	VIC (rural)	Women attending general practices	Cross-sectional survey	Swab	n.d.	F	15–35	n.r.	67	9	13.4 (6.3, 24.0)*
Toyne [30]	ACT	Women attending general practices	Cross-sectional survey	Swab	n.d.	F	17–39	2002–2003	353	4	1.1 (0.3, 2.9)
Bowden [31]	ACT	Women attending general practices	Cluster randomised controlled trial	Swab/ urine	6.9 in intervention; 4.5 in control	F	16–39	2004–2005	1593	69	4.3 (3.4, 5.5)
							(16–19)		196	10	5.1 (2.5, 9.2)
							(20–25)		576	37	6.4 (4.6, 8.7)
							(26–30)		425	19	4.5 (2.7, 6.9)
							(31–39)		396	3	0.7 (0.2, 2.2)
Hince [32]	WA (Perth)	Men attending general practices	Cross-sectional survey	Urine	63–100	M	15–29	2007–2008	383	14	3.7 (2.0, 6.1)**
Bilardi [33]	VIC (urban and rural)	Women attending general practices	Cluster randomised controlled trial	Any	6.2–11.5	F	16–24	2008–2009	843	66	7.8 (6.1, 9.9)*
Walker [34]	Australia-wide	Women attending primary health care clinics	Cross-sectional survey	Swab	66	F	16–25	2007–2008	738	25	3.4 (1.5, 5.3)

Studies are presented in order of publication year and author. * Confidence intervals calculated by authors. ** Re-calculated confidence intervals differ from those reported. ^A^ Median. ACT, Australian Capital Territory; Bris, Brisbane; F, female; GP, general practice, general practitioner; M, male; Melb, Melbourne; NA, not applicable; n.d., not determined; n.r., not reported; NSW, New South Wales; QLD, Queensland; VIC, Victoria; WA, Western Australia. Participant numbers reflect numbers from which epidemiological data was calculated, with sub-group numbers (e.g. by age or year) in brackets.

Where both male and female participants were included (30 studies), a substantial portion (16 studies) did not report these data separately, or failed to report by sex for some categories. These data were excluded from further meta-analysis.

Where reported, the most commonly utilised sample was first-void urine for both men and women; however, other samples included urethral, rectal and pharyngeal swabs for men; and endocervical swabs, self-obtained lower vaginal swabs, self-inserted tampons, and fallopian tube washings for women.

### General practice and community settings

Eighteen studies (18 papers; 1 abstract) reported on chlamydia prevalence estimates from studies conducted in GP or community-based settings (Table 1) [16-34]. Seven reported on prevalence measured in GP clinics [28-34], while other studies recruited participants from community-based settings including sporting clubs (3) [21,22,24,25], university or high school campuses (5) [17-20,23], festivals (2) [23,26], backpacker hostels (2) [23,27], and a defense force unit (1) [23]. Only one study utilised a population-based sample of young women recruited from a telephone directory [16]. Participation rates were highest (>80%) in studies conducted in sporting clubs [21,22,24,25], and lowest in two GP-based RCTs of chlamydia testing interventions (<11.5%) [31,33].

In community-based settings, reported prevalence estimates were 3.9–8.5% for men and 7.4% for women recruited from sporting clubs [21,22,24,25], 3.5–7.7% among male and female backpackers [23,27], and 0.0–5.3% for young people in educational settings [17-20,23]. The only population-based study reported a prevalence of 3.7% among sexually active 18–24-year-old women [16], with no population-based data available for men.

In general practice, chlamydia prevalence for women ranged from 1.1–13.4%, although the highest and lowest estimates were from studies with either a small sample size [29], or a higher proportion of older women [29]. The most recent cross-sectional surveys, one in men [32] and one in women [34], had similar participation rates of over 60% and reported similar prevalence estimates for males and females (3.7% and 3.4%, respectively). Prevalence was higher in the two RCTs, at 5.1–7.8% among women aged 16–25 years; however, response rates were low (<11.5%).

The majority of GP and community-based studies (14) provided prevalence estimates for those aged 30 years or less, with nine studies providing estimates for those aged 25 years or less, and just three providing age-stratified data [16,27,31]. For women, one clinic-based [31] and one community-based study [16] reported higher prevalence among women under 25 years, compared with older women: 6.1% and 3.7% versus 5.5% and 1.7%, respectively. A third study measured prevalence in both male and female International backpackers [27] and reported a higher chlamydia prevalence in women aged over 25 years (7.3%), compared with younger women (3.0%); with the trend reversed in men (1.6% versus 3.6%). Only one study estimated prevalence over time, with no trend evident [18].

**Figure 2:**
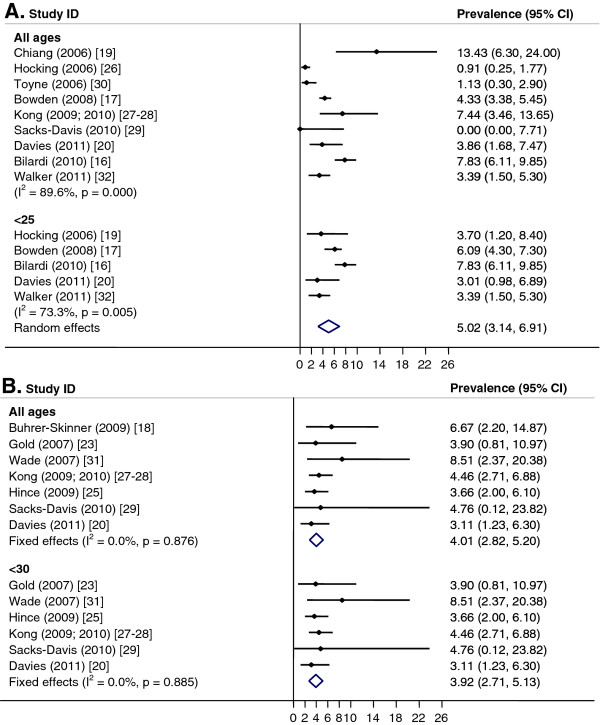
**Chlamydia in general practice and community settings.** Meta-analysis of chlamydia prevalence estimated in general practice and community settings. **A**. Females. **B**. Males.

Pooled prevalence estimates for women aged <25 years or for women of any age group could not be calculated due to significant heterogeneity between studies (I^2^ > 75%, p < 0.01). However, if studies conducted prior to 2006 were excluded, the pooled prevalence of women aged <25 years was 5.0% (CI: 3.1, 6.9; I^2^ = 73%, p < 0.01) (Figure 2A) and if the two RCTs with low response rates were excluded [31,33], the pooled prevalence for under 25 year old women was 3.3% (CI: 1.9, 4.8; I^2^ = 0%, p = 0.96) and 2.6% (CI: 1.0, 4.1; I^2^ = 72%, p < 0.01) for women of all ages. For men, a pooled estimate of 3.9% (CI: 2.7, 5.1; I^2^ = 0%, p = 0.88) was calculated for men aged <30 years and 4.0% (CI: 2.8, 5.2; I^2^ = 0%, p = 0.88) for all ages (Figure 2B). There was only one study for <25-year-old men conducted in the period from 2006 onward and this reported a prevalence of 3.7% (CI: 0.4, 6.9) [27]; all studies for men <30 years were conducted post-2005. Pooled estimates by setting were not calculated because of significant heterogeneity between studies (I^2^ >75%, p < 0.01). However, if studies conducted prior to 2006 and the two RCTs with low response rates were excluded, the pooled prevalence was higher in GP clinics than in community-based studies (3.5%; CI: 2.1, 4.9; I^2^ = 0%, p = 0.85, versus 2.9%; CI: 2.0, 3.8; I^2^ = 0%, p = 0.47). 

### Sexual health centres, youth health services and other clinical settings

There were 24 studies (23 papers; 2 abstracts) reporting chlamydia prevalence estimates in clients attending sexual health or family planning clinics (14 studies) [34-48], youth centres (8) [18,23,28,45,49-53] and other clinical settings (4) [45,54-56] ( Additional file 1).

For studies conducted at sexual health clinics, the majority (7) were retrospective audits of patient records [36,38,39,41,43,44,48,57], five were cross-sectional surveys [34,37,40,42,47] and two were sentinel surveillance reports [45,46]. Sample sizes varied greatly, from 175 [42] to 59,720 [46]. Six studies provided estimates for women only [34,37,40,42,43,47], three for men only [35,41,48], and four for both males and females [36,38,39,45,46]; one study did not differentiate between males and females [44]. Prevalence estimate ranges were 5.3–13.4% for males and 0.6–13.0% for females, although the study with a prevalence of 0.6% excluded women <18 years and those with pelvic inflammatory disease [42]. Where age-stratified estimates were available, prevalence tended to be highest among those <25 years for both males and females [37-39,43,47,48].

Three studies reported on prevalence trends over time in sexual health clinics [43,44,48]. Two comprehensive analyses found that chlamydia prevalence increased by about 12% per year (from 4.2% in 2003 to 6.7% in 2007) among females [43] and by 3% per year (from 5.8% in 2002 to 8.0% in 2009) among heterosexual males [48] after adjusting for changes in patient demographics, clinical presentation or sexual behaviour over time.

Two cross-sectional surveys and one sentinel surveillance study compared chlamydia prevalence in sexual health and family planning clinics across multiple geographic locations. A study of women attending family planning clinics found a higher prevalence at an inner city clinic: 4.8% *versus* 1.7% at a suburban clinic [37]. Younger women (<25 years) attending the inner-city clinic had a higher prevalence (6.2%), whereas the prevalence for this age group at the suburban clinic was 1.7%. Similarly, higher prevalence estimates in inner-city clinics compared with suburban clinics were reported by Bateson and colleagues [40] (9.7% versus 3.1%).

For the eight studies that determined prevalence at youth centres [23,28,45,49-53], prevalence estimates ranged from 0–20.0%. One study reported increased prevalence from one year to the next (2.7% in 2006/07 *vs.* 11.3% in 2007/08); however, the increase likely reflected a change in the mode of operation from an appointment-based to drop-in service [53]. Sample sizes in this setting were also frequently low, and half did not report data for males and females separately [28,49,52,53].

In non-primary care clinical settings, the chlamydia prevalence for women attending a colposcopy clinic was reported to be higher in women aged 25 years or less (5.8%) compared with older women (0.9%) [55]. A prevalence of just 0.2% was found for women attending a hospital in vitro fertilisation (IVF) service for investigation of infertility [54]; however, higher estimates for hospital-based studies of 3.1% for women [45] and 5.5% for men and women [56] were also reported. These divergent estimates were possibly due to age differences in study participants, although the precise age range of participants was not available for two of the studies [45,54].

**Figure 3:**
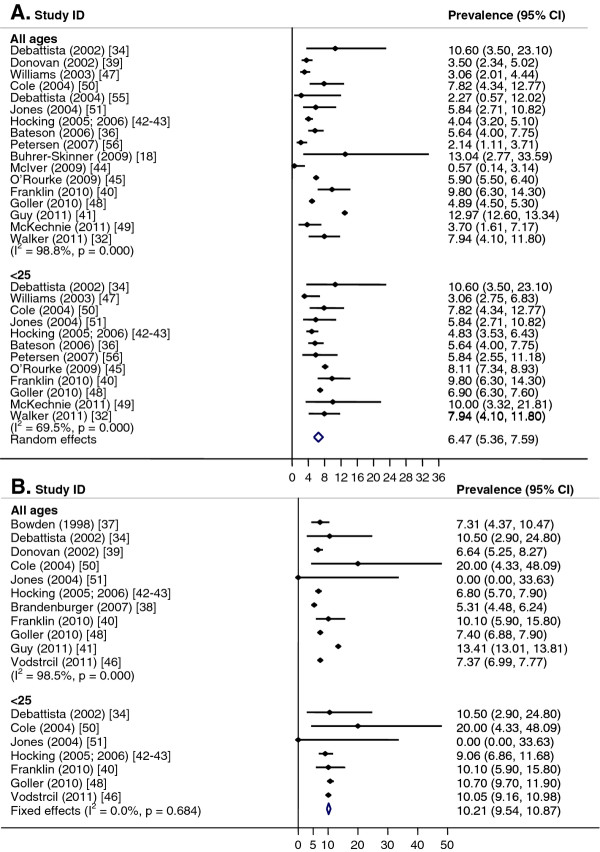
**Chlamydia in sexual health clinics, youth services and other clinical settings.** Meta-analysis of chlamydia prevalence estimated in sexual health clinics, youth services and other clinical settings. **A**. Females. **B**. Males.

Meta-analysis was performed for males and females separately by age group (Figure 3). The pooled prevalence for women <25 years was 6.2% (CI: 5.1, 7.4; I^2^ = 73%, p < 0.01). It was not possible to calculate the pooled prevalence for women of all ages because of significant heterogeneity between studies (I^2^ = 99%, p < 0.01) (Figure 3A). When analysed by setting, there was significant heterogeneity for studies conducted in sexual health or family planning clinics, but for women attending youth centres, the pooled prevalence was 6.8% (CI: 3.8, 9.7; I^2^ = 0%, p = 0.51) for women <25 years and 7.0% (CI: 4.1, 9.9; I^2^ = 0%, p = 0.59) for women of all ages. Within other clinical settings, the pooled prevalence was 2.1% (CI: 0.9, 3.4; I^2^ = 0%, p = 0.97) for women of all ages.

For men, the pooled prevalence for men of all ages across all study sites was not calculated due to heterogeneity (I^2^ = 98.8%, p < 0.01); however, the pooled prevalence was 10.2% (CI: 9.5, 10.9; I^2^ = 0%, p = 0.41) for men <25 years (Figure 3B). In sexual health/family planning clinics, the pooled prevalence was 9.9% (CI: 9.1, 10.8; I^2^ = 0%, p = 0.45) for men <25 years; it was not possible to calculate the pooled prevalence for men of all ages attending sexual health/family planning clinics because of significant heterogeneity between studies (I^2^ = 99%, p < 0.01). In youth centres, the pooled prevalence was 8.7% (CI: 0, 28.2; I^2^ = 50%, p = 0.16) among men <25 years.

### Pregnant women

Eleven studies (10 papers; 1 abstract) reported prevalence estimates in pregnant women, from a range of urban and rural/remote locations ( Additional file 2) [58-68]. Sample sizes ranged from 70 [59] to 1175 [58] participants, and participation rates in most studies were high (52–99.8%).

Reported chlamydia prevalence estimates were diverse, ranging from 2.8–14.4%, with prevalence highest among young and/or Indigenous women. In studies with age-stratified data, all reported higher prevalence estimates in women <25 years (4.5–22.0%), compared with participants of all ages (2.7–14.4%) [62-64]. Three studies of women aged 20 years or younger reported estimates of 5.7–13.7% [60,61,65], and three further studies where age was not specified reported prevalence estimates of 2.8% [59], 3.9% [66] and 12.3% [68]. A large study conducted in public hospital antenatal clinics in Victoria reported a prevalence of 3.2% among women <25 years [67].

For pregnant Indigenous women, rates of infection ranged from 2.9–14.4% [59,63,64,68]. A comparison of prevalence in Indigenous versus non-Indigenous women reported higher estimates in Indigenous women: 9.1% compared with 2.7% overall [64]. One study reporting age-stratified data found a prevalence of 14.4% overall, with higher prevalence in teenagers (32.6%) [63]. This is also higher than the two hospital-based teen studies, despite both having high proportions of Indigenous participants: 30.3% [60] and 74.6% [61].

**Figure 4:**
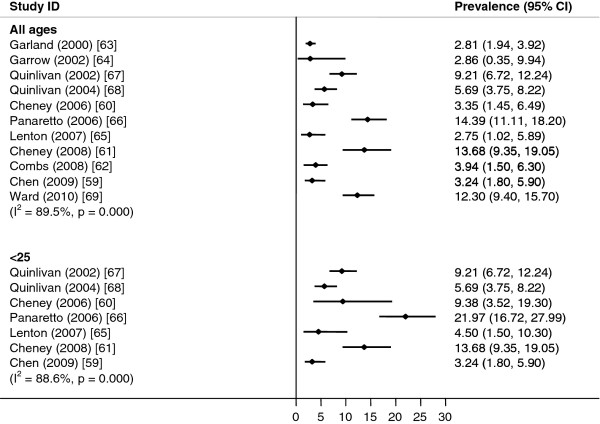
**Chlamydia in pregnant women.** Meta-analysis of chlamydia prevalence estimated in pregnant women.

It was not possible to calculate pooled prevalence estimates because of significant heterogeneity between the studies (I^2^ = 89.5%, p < 0.01), which persisted when the studies were stratified by age (<25 years, I^2^ = 88.6%, p < 0.01; Figure 4).

### Indigenous Australians

Prevalence estimates for Indigenous Australians were reported in 16 reviewed studies (17 papers; 1 abstract) [18,23,44,59,63,64,68-78] ( Additional file 3). Sample sizes varied from 20 [23] to 2817 [72] and while participation rates were reported in only seven of the studies, four cited rates of over 80%. The majority of studies were conducted in rural or remote areas (5) [59,69,71-73,79] or regional centres (6) [23,63,68,74,76-78]. Only three studies were conducted in large capital cities [18,23,44], and one sentinel surveillance study reported data from both urban and rural locations [77].

Estimates ranged from 4.8–14.4% for women, and 8.6–16.3% for men. Three studies only reported combined prevalence estimates for men and women (4.4–15%). The highest reported estimates were in younger age groups. Two studies reported age-stratified data, with prevalence very high in both male and female teens (males 13.0–18.8%; females 17.4–34.3%), and then decreasing with age [72,77]. A further study of Indigenous youths reported high estimates of 21.0% for males and 11.8% for females aged 15–18 years [18]; while pregnant Indigenous teens recorded prevalence estimates ranging from 2.9% [59] to 32.6% [63].

In studies that compared Indigenous with non-Indigenous individuals, three of four studies reported higher prevalence estimates for Indigenous study participants [23,63,64]. One study found that, when considered separately, women identifying as Torres Strait Islanders had a prevalence of 9.7%, whereas Aboriginal women had a prevalence of just 3.5%, lower than for non-Indigenous women in the study (3.8%) [63]. A single study conducted in a large urban sexual health clinic found a lower prevalence in Indigenous (6.7%) compared with non-Indigenous clients (9.4%), although this was not a significant difference [44].

Just one study reported prevalence estimates over time [69,70], finding that prevalence decreased for both men and women over the first study period (1998–2000), from 8.8% to 7.2% in men, and from 9.1% to 7.2% in women. However, this was linked to the introduction of a sexual health intervention, and the falls were not significant [69]. The odds of testing positive for chlamydia decreased significantly by 12% per year (CI: 8, 16) between 1996 and 2002, but no further reduction was observed between 2003 and 2006 [70].

**Figure 5:**
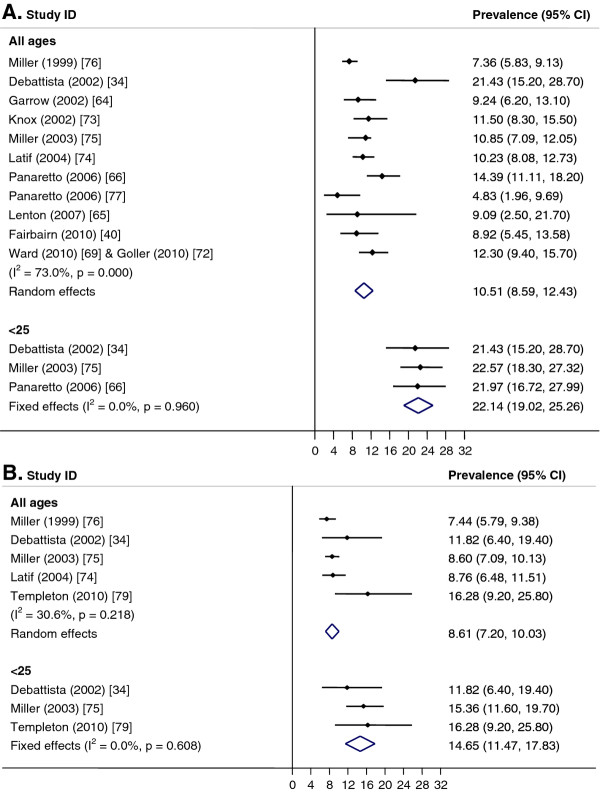
**Chlamydia in Indigenous Australians.** Meta-analysis of chlamydia prevalence estimated in Aboriginal and Torres Strait Islander individuals. **A**. Females. **B**. Males.

Pooled prevalence estimates were higher for women than for men and higher among those aged <25 years (Figure 5). Among women, the pooled prevalence for those <25 years was 22.1% (CI: 19.0, 25.3; I^2^ = 0%, p = 0.96) and across all ages, 10.5% (CI: 8.6, 12.4; I^2^ = 73%, p < 0.01). For men, the pooled prevalence for <25-year-olds was 14.7% (CI: 11.5, 17.8; I^2^ = 0%, p = 0.61) and across all ages, 8.6% (CI: 7.2, 10.0; I^2^ = 31%, p = 0.22).

### Men who have sex with men (MSM)

Sixteen studies (19 papers) were identified that measured chlamydia prevalence in MSM [38,39,44-46,48,79-91] ( Additional file 4). Sample sizes ranged from 80 [82] to 11,777 [91], and settings included sexual health centres (11 studies) [38,39,44-46,48,79,81,84,85,89-91], hospitals and other clinical settings (3) [85,86,90], and male-only entertainment or sex-on-premises venues (SOPVs; 4) [79-81,83]. One study involved community-based cohorts of HIV-positive and HIV-negative MSM [87,88]. Where reported, participation rates were high in clinical settings (77–85%), but much lower in SOPVs (24–50%).

Overall, 12 studies reported prevalence estimates for urethral infection, 10 for rectal infection, and 10 for pharyngeal infection, while five did not provide site-specific estimates. The prevalence of rectal infection was consistently more than 30% higher than urethral infection and ranged from 4.0% among HIV-positive men attending HIV clinics [90] to 14.7% among men attending a sexual health clinic [84]. Urethral infection prevalence ranged from 1.0% [86] to 5.0% [84]. Estimates for pharyngeal infection were low, ranging from 0.0% in both clinical and SOPV settings [80,82,90] to 2.7% [85]. A large well-conducted cohort study found the prevalence of rectal and urethral chlamydia at the time of recruitment to be higher among HIV-positive compared with HIV-negative men (5.9% versus 4.4% and 0.9% versus 2.2%), although this was not statistically significant [87,88].

**Figure 6:**
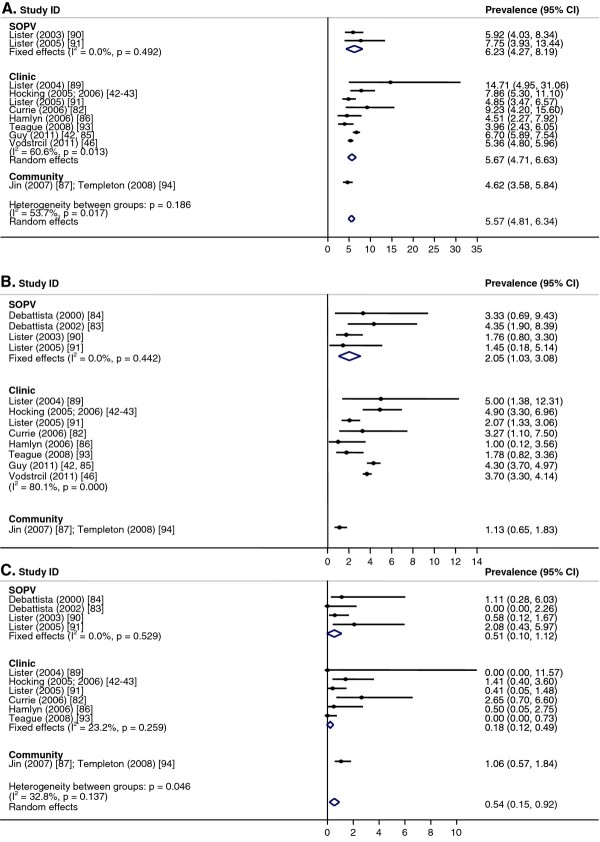
**Chlamydia in men who have sex with men (MSM).** Meta-analysis of chlamydia prevalence estimated in men who have sex with men (MSM). **A**. Rectal. **B**. Urethral. **C**. Pharyngeal.

The pooled prevalence for rectal chlamydia was 5.6% (CI: 4.8, 6.3; I^2^ = 54%, p = 0.02) and was similar between men tested in SOPVs (6.2%: CI: 4.3, 8.2; I^2^ = 0%, p = 0.49) and in clinics (5.7%, CI: 4.7, 6.7; I^2^ = 61%, p = 0.01) (Figure 6A). It was not possible to calculate an overall pooled prevalence for urethral chlamydia because of heterogeneity between studies (I^2^ = 86%, p < 0.01) (Figure 6B). This was also the case for clinic-based urethral chlamydia prevalence (I^2^ = 80%, p < 0.01). The pooled prevalence for urethral chlamydia among men tested at SOPVs was 2.1% (CI: 1.0, 3.1; I^2^ = 0%, p = 0.44). For pharyngeal chlamydia (Figure 6C), the pooled prevalence across settings was 0.5% (CI: 0.2, 0.9; I^2^ = 33%, p = 0.14), and was similar between SOPVs (0.5%; CI: 0, 1.1; I^2^ = 0%, p = 0.53) and clinical settings (0.3%; CI: 0, 0.8; I^2^ = 23%, p = 0.26).

### High-risk populations

Twelve papers were identified that estimated chlamydia prevalence in potentially high-risk populations [18,38,44,60,78,89,92-97] ( Additional file 5). Five measured chlamydia prevalence in sex workers (legal and illegal) [38,44,92-94], four in individuals in prison or juvenile detention [18,78,95,96], two in drug users [60,97], and one in STI contacts [89]. Participation rates, where reported, were high (49–87%), and sample sizes varied from 86 [78] to 1766 [93].

Prevalence in sex workers ranged from 1.2–8.3%. Three of the five studies did not differentiate between male and female sex workers, although all had predominantly female participants [38,92,93]. Brothel workers had lower prevalence estimates in two studies, compared with street sex workers [92,93]. However, a study of illegal brothel sex workers in Western Australia found a low prevalence of 2.7% [94]. Two clinical audits conducted at sexual health centres found divergent estimates of 3.3% [38] and 7.8% [44] for female sex workers, and 11.1% for male sex workers [44].

For prisoners, a study by Mak and colleagues [95] reported that 3% of male prisoners tested positive for chlamydia upon admission to prison. A larger study conducted across male and female prisons in both metropolitan and regional locations recorded an overall prevalence of 7.3% [96]. For the juvenile detainees, chlamydia prevalence for females was measured at over 20 per cent in two separate studies [18,96], higher than for male juveniles (range 2.0–16.3%).

In a sample of injecting drug users, 6% tested positive for chlamydia [97]; and in pregnant teens, chlamydia was more prevalent in multi-drug– (25.8%) and marijuana-users (9.7%), compared with non-users (7.7%). The highest prevalence (33.1%) was reported for clients attending a sexual health centre as contacts of chlamydia, non-gonococcal urethritis and PID [89].

## Discussion

We found that chlamydia prevalence estimates were highly variable, with rates generally higher among young Australians, Indigenous Australians, and MSM. Other high-risk populations, including youth clinic attendees, pregnant teenagers and prison inmates, were also found to have higher prevalence estimates. This review builds considerably on the earlier review by Vajdic et al. [12], and a key strength is that studies were limited to those that used the more sensitive and specific NAAT testing methods.

Measuring the true prevalence of chlamydia in a community is a challenging task, with non-population-based studies frequently suffering from sampling bias and low participation rates [98,99]. Studies utilising population-based data on chlamydia prevalence are rare, both in Australia and the UK [12,100], and this review identified just one survey that recruited participants from the general population. This study, of women recruited from the telephone directory, was limited by its small sample size and low participation rate (43%) [16]. Both community-based and clinic-based sampling is subject to participation bias: community-based sampling often relies on convenience sampling, where participants are able to self-select; and clinic-based sampling is likely to bias towards symptomatic and higher risk participants, and towards more health care–seeking, and therefore well-educated, Australian-born participants [34]. This can also be true of population-based studies [16]. Sexual health centre studies can be particularly susceptible to bias towards symptomatic and high-risk groups, although as a research setting, they can provide large sample sizes at low costs, especially through clinic audits.

Community-based studies are important for estimating chlamydia prevalence as notification rates are known to under-estimate infection levels, as they are highly influenced by testing rates. The only population-based estimate identified in this review reported a prevalence of 3.1% among all 18 to 24 year old women in 2003–2004 [16]; no similar study has been conducted in men. This would translate into over 10,500 cases of chlamydia among Victorian women aged 15–24 years in 2004 (and 10,900 cases in men of the same age), which is considerably higher than the 7694 cases notified among both men and women of any age in Victoria that year [2], highlighting that notification data considerably underestimate of the true number of individuals infected.

A key factor that limits the conclusions that can be drawn from the studies included in the review is the high level of heterogeneity both within and between the populations studied. In most key populations reviewed, pooled prevalence estimates could not be calculated, even after stratifying data by age and sex of individuals tested. This was particularly true for female data. Although we calculated pooled prevalence estimates within the different population sub-groups, it is important that these be interpreted with caution and only indicative of the true prevalence.

The high level of variability between studies also prevented prevalence trends over time to be assessed, despite the review period spanning almost 15 years. Only seven individual studies determined time trends [18,36,43,44,48,69], three finding a statistically significant increase in prevalence over time [36,43,48]; and two reporting no change over time [18,44]. One study in an Indigenous community found a decrease over time, however, this was associated with an STI intervention to increase testing rates [69]. Despite the variability, the studies by Vodstrcil et al. [48] and O’Rourke et al. [43] – both conducted in sexual health clinics – provide evidence to support an increasing chlamydia prevalence among heterosexual men and women in Australia, because these studies had large sample sizes and adjusted for changes in sexual behaviour over time. Both Australian and international data show that sexual risk behaviour has changed over the last decade with increasing numbers of sex partners reported by young adults. The National Survey of Sexual Attitudes and Lifestyles, a sexual behaviour survey conducted in the United Kingdom in 1990 and again in 2000 [101] showed that the number of heterosexual partners in the preceding five years increased significantly for both sexes. Australian data show that age at first sexual intercourse has decreased, with women aged 16–24 years reporting a median age of 16 at first sexual intercourse compared with 19 for women aged 50–59 years [102].

Similar to previous systematic reviews in both the UK and Australia [12,100], we found that the study setting influenced the prevalence estimates reported; however, significant heterogeneity again hampered comparisons in most cases. For men, prevalence was higher in sexual health and family planning clinics compared with GP and community-based settings. This was similar for women aged <25 years; however, this difference was not statistically significant. GP-based studies, similar to those conducted by Vodstrcil et al. [48] and O’Rourke et al. [43] in sexual health centres, would greatly enhance our ability to compare between these clinical settings. Between-setting comparisons are especially fraught for men; only one study conducted in general practice reported prevalence among men alone [32], and no studies reported chlamydia prevalence in heterosexual men from a population-based sample.

Where studies reported age-based estimates, younger participants had higher prevalence estimates than older participants. These data are consistent with the national notification data that show that notification rates are highest among those <25 years [2] and are also consistent with sexual behaviour data which show that numbers of sexual partners are highest in these younger age groups [103]. We also found high rates among disadvantaged youth and young people attending youth clinics. These findings of increased chlamydia among young men and women echo those findings in overseas prevalence studies [98,99].

Chlamydia notification rates over the past 15 years have been consistently higher in women compared with men; however, this did not emerge as a robust trend in this review. Higher female notification rates can probably be attributed to differences in chlamydia testing rates. In Australia, recent Medicare data indicates that about 12–13% of sexually active young women and 3–4% of young men are tested for chlamydia each year [11]. As chlamydia testing rates increase in Australia, notification data will be able to provide a better estimate of the population prevalence of chlamydia.

We found that prevalence estimates were comparable among heterosexual men and women; however, the picture is neither complete nor consistent. In the general practice setting, no studies directly compare prevalence between men and women; and in sexual health clinics, prevalence tended to be higher among men. This is probably because men are more likely to attend a sexual health centre due to the presence of urethral symptoms [104]. Curiously, fifteen studies were identified that did not report male and female data separately, thereby excluding the data from calculations of pooled prevalence estimates. A number of recent studies reporting chlamydia prevalence in men attending sporting clubs [21,22,24,25] and general practices [32] have started to address the predominance of female studies, which has been previously noted [12]; however, there remains a need for additional studies that directly compare men and women in community and clinical settings.

Similar discrepancies between notification data and population-based prevalence surveys have also been observed in the USA. In 2010, the notification rate reported to the Centers for Disease Control and Prevention was 2.6 times higher for women (610.6 per 100,000 population) than for men (233.7 per 100,000) [105]; rates in 2002 differed by almost four-fold between the sexes. By comparison, prevalence estimates reported in the National Health and Nutrition Examination Survey (NHANES) conducted between 1999–2002 were similar in women (2.5%; CI: 1.8, 3.4) and men (2.0%; CI: 1.6–2.5) [106]. Although the prevalence was twice as high in women aged 14–19 years (4.6%) compared to men (2.3%), the trend was reversed in the 20–29 year age group, where more men were infected (3.2%) than women (1.9%) [106]. These data underscore the fact that at low testing rates, notification data do not provide a full picture of the prevalence of chlamydia infection in the community.

In contrast to heterosexual men, several studies explored chlamydia prevalence among MSM, with most providing estimates from multiple anatomical sites (Additional file 4). In line with data from the UK and USA [107,108], prevalence was highest in rectal swabs compared with urethral samples, and lowest in pharyngeal swabs. This highlights the importance of rectal chlamydia screening in MSM and the need to include both urethral and rectal sampling when conducting chlamydia prevalence surveys in this population group as recommended in national guidelines [109]. Unfortunately, Australian national notification data do not include site of infection nor sexual orientation, thereby reducing our ability to monitor trends in this population group over time.

The key gaps identified by Vajdic et al. [12] still remain today, including a need for population-based data for young men and women and systematically collected serial sentinel data with which trends in chlamydia prevalence over time can be monitored, particularly within the nationally identified target risk groups (young men and women, MSM and Indigenous Australians) [110]. The further advances in information technology including improvements in medical records software, the development of data extraction software [111] and data linkage [112], will facilitate the collection of standardised and detailed socio-demographic, behavioural and clinical data (including presence or absence of chlamydia-related symptoms) from sentinel sites. This will allow trends to be evaluated over time within different risk groups, adjusting for any changes in behavioural data and clinical presentation. The Australian Collaboration for Chlamydia Enhanced Sentinel Surveillance (ACCESS; http://www.access-study.org) [44] and the Victorian Primary Care Network for Sentinel Surveillance (VPCNSS) [45] are both important sentinel surveillance projects that collect detailed demographic, clinical and behavioural data with which trends in risk groups can be monitored over time. It is vital that such surveillance systems continue to be funded. Further, the Australian Chlamydia Control Effectiveness Pilot (ACCEPt; http://www.accept.org.au), a randomised controlled trial of chlamydia testing in general practice, is collecting chlamydia testing data from about 250 GP clinics across Victoria, New South Wales, Queensland and South Australia and will provide trends in chlamydia positivity over time among young men and women.

## Conclusions

This comprehensive systematic review identified 76 studies reporting prevalence data for individuals tested for anogenital or pharyngeal chlamydia and provides an up-to-date summary of the underlying burden of chlamydia in Australian populations. The review highlights that the burden of chlamydia in Australia is greatest among young adults, Indigenous populations and MSM and identifies important gaps in the surveillance and monitoring of chlamydia infection in Australia. Given that that the Australian Government is currently pilot testing chlamydia screening as a national program and State Governments continue to fund chlamydia control activities, it is vital that good sentinel surveillance systems continue.

## Abbreviations

ACT, Australian capital territory; Bris, Brisbane; CI, 95% confidence interval; GP, General practice, general practitioner; Melb, Melbourne; MSM, Men who have sex with men; NA, Not applicable; NAAT, Nucleic acid amplification test; n.d., Not determined; n.r., Not reported; NSW, New South Wales; NT, Northern Territory; PID, Pelvic inflammatory disease; QLD, Queensland; SA, South Australia; SOPV, Sex on premises venue; VIC, Victoria; WA, Western Australia.

## Competing interests

The authors declare that they have no competing interests.

## Authors’ contributions

JSH, DL and DCN conceived of the study, and participated in its design and coordination. DL and DCN conducted the literature search and systematic review. JSH performed the meta-analysis. DL, DCN, RJG, HA, MYC, CKF and JSH contributed to drafting the manuscript. All authors read and approved the final manuscript.

## Pre-publication history

The pre-publication history for this paper can be accessed here:

http://www.biomedcentral.com/1471-2334/12/113/prepub

## Supplementary Material

Additional file 1: **Studies reporting chlamydia prevalence data, identified in sexual health clinics, youth services and other clinical settings.** Studies are presented in order of publication year and author. * Confidence intervals calculated by report authors. ** Re-calculated confidence intervals differ from those reported. ^A^ Median. F, female; M, male; Melb, Melbourne; NA, not applicable; n.d., not determined; n.r., not reported; NSW, New South Wales; NT, Northern Territory; QLD, Queensland; VIC, Victoria; WA, Western Australia. Participant numbers reflect numbers from which epidemiological data was calculated, with sub-group numbers (e.g. by age or year) in brackets. (DOC 89 kb) Click here for file

Additional file 2: **Studies reporting chlamydia prevalence data, identified in pregnant women.** Studies are presented in order of publication year and author. * Confidence intervals calculated by authors. ** Re-calculated confidence intervals differ from those reported. ^A^ Median. Melb, Melbourne; NA, not applicable; n.d., not determined; n.r., not reported; NSW, New South Wales; QLD, Queensland; VIC, Victoria; WA, Western Australia. Participant numbers reflect numbers from which epidemiological data was calculated, with sub-group numbers (e.g. by age or year) in brackets. (DOC 41 kb) Click here for file

Additional file 3: **Studies reporting chlamydia prevalence data, identified in Indigenous Australians.** Studies are presented in order of publication year and author. * Confidence intervals calculated by authors. ** Re-calculated confidence intervals differ from those reported. ^A^ Median. Bris, Brisbane; F, female; GP, general practice, general practitioner; M, male; Melb, Melbourne; NA, not applicable; n.d., not determined; n.r., not reported; NSW, New South Wales; NT, Northern Territory; QLD, Queensland; SA, South Australia; WA, Western Australia. Participant numbers reflect numbers from which epidemiological data was calculated, with sub-group numbers (e.g. by age or year) in brackets. (DOC 53 kb) Click here for file

Additional file 4: **Studies reporting chlamydia prevalence data, identified in men who have sex with men.** Studies are presented in order of publication year and author. * Confidence intervals calculated by report authors. ** Re-calculated confidence intervals differ from those reported. ^A^ Median. ACT, Australian Capital Territory; HIV+, HIV-positive; HIV–, HIV-negative; Melb, Melbourne; NA, not applicable; n.d., not determined; n.r., not reported; NSW, New South Wales; Ph, Pharynx; QLD, Queensland; R, rectum; SHC, sexual health centre; U, urethra; VIC, Victoria. Participant numbers reflect numbers from which epidemiological data was calculated, with sub-group numbers (e.g. by age or year) in brackets. (DOC 54 kb) Click here for file

Additional file 5: **Studies reporting chlamydia prevalence data, identified in high-risk populations.** Studies are presented in order of publication year and author. * Confidence intervals calculated by authors. ** Re-calculated confidence intervals differ from those reported. ^A^ Median. Bris, Brisbane; broth, brothel; CBD, central business district; F, female; M, male; Melb, Melbourne; NA, not applicable; n.d., not determined; n.r., not reported; NSW, New South Wales; QLD, Queensland; st, street; WA, Western Australia. Participant numbers reflect numbers from which epidemiological data was calculated, with sub-group numbers (e.g. by age or year) in brackets. (DOC 48 kb) Click here for file
