# Supplementary material for: The prevalence of Chlamydia trachomatis infection in Australia: a systematic review and meta-analysis
Source: BMC Infect Dis. 2012 May 14;12:113. doi: 10.1186/1471-2334-12-113 (PMC3462140; doi:10.1186/1471-2334-12-113)
Supplement: Additional file 1: — Studies reporting chlamydia prevalence data, identified in sexual health clinics, youth services and other clinical settings. Studies are presented in order of publication year and author. * Confidence intervals calculated by report authors. ** Re-calculated confidence intervals differ from those reported. A Median. F, female; M, male; Melb, Melbourne; NA, not applicable; n.d., not determined; n.r., not reported; NSW, New South Wales; NT, Northern Territory; QLD, Queensland; VIC, Victoria; WA, Western Australia. Participant numbers reflect numbers from which epidemiological data was calculated, with sub-group numbers (e.g. by age or year) in brackets. (DOC 89 kb) [file 1471-2334-12-113-S1.doc]

## Additional File 1 - Studies reporting chlamydia prevalence data, identified in sexual health clinics, youth services and other clinical settings

Studies are presented in order of publication year and author. ***** Confidence intervals calculated by report authors. ** Re-calculated confidence intervals differ from those reported. ^A^ Median. F, female; M, male; Melb, Melbourne; NA, not applicable; n.d., not determined; n.r., not reported; NSW, New South Wales; NT, Northern Territory; QLD, Queensland; VIC, Victoria; WA, Western Australia. Participant numbers reflect numbers from which epidemiological data was calculated, with sub-group numbers (e.g. by age or year) in brackets.

| **Study** | **Location** | | | **Participants** | | **Study design** | **Specimen type** | **Response rate (%)** | **Sex** | **Age (years)** | **Study period** | **Tested  (n)** | **Positive (n)** | **Prevalence**  **% (95% CI)** |
| --- | --- | --- | --- | --- | --- | --- | --- | --- | --- | --- | --- | --- | --- | --- |
| **Sexual Health and Family Planning Centres** | | | | |  |  |  |  |  |  |  |  |  |  |
| Bowden (1998) [35] | NT (Darwin) | | | Male sexual health clinic clients | | Clinical audit | Urine/swab | NA | M | n.r. | 1996–1997 | 301 | 22 | 7.3 (4.4, 10.5)* |
| Donovan (2002) [36] | NSW (Sydney) | | | Heterosexual men and women attending a sexual health clinic | | Clinical audit | F: swab;  M: urine | NA | F  M | 28–30^A^  25–26 ^A^ | 2000 | 800  1114 | 28  74 | 3.5 (2.3, 5.0)*  6.6 (5.3, 8.3)* |
| Williams (2003) [37] | VIC (Melb) | | | Women attending Family Planning clinics | | Cross-sectional survey | Urine | 77 | F | ≥13  (<25)  (25+) | n.r. | 851  447  385 | 26  20  6 | 3.1 (0.1, 4.5)*  4.5 (2.8, 6.8)*  1.6 (0.6, 3.4)* |
| Hocking (2005) [38]; Hocking (2006) [39] | VIC (Melb) | | | Heterosexual men and women attending a sexual health clinic | | Clinical audit | Urine/swab | NA | F  M | All  (<20)  (20–24)  (25–29)  (30–34)  (35–39)  (40+)  All  (<20)  (20–24)  (25–29)  (30–34)  (35–39)  (40+) | 2002–2003 | 1782  209  701  450  197  101  122  2028  72  513  505  357  202  379 | 72  12  32  18  5  3  1  138  7  46  29  27  11  17 | 4.0 (3.2, 5.1)  5.7 (3.0, 9.8)  4.6 (3.1, 6.4)  4.0 (2.4, 6.2)  2.5 (0.8, 5.8)  3.0 (0.6, (8.4)  0.8 (0.0, 4.4)  6.8 (5.5, 7.7)*  9.7 (4.0, 19.0)  9.0 (6.6, 11.8)  5.7 (3.9, 8.1)  7.6 (5.0, 10.8)  5.4 (2.7, 9.5)  4.5 (2.6, 7.1) |
| Bateson (2006) [40] | NSW (Various) | | | Women attending family planning clinics | | Cross-sectional survey | Urine | 67 | F | 16–24 | 2004 | 621 | 35 | 5.6 (4.0, 7.8)** |
| Branden-burger (2007) [41] | ACT (Can-berra) | | | Men attending a sexual health clinic | | Clinical audit | Urine/swab | NA | M | n.r. | 2003–2005 | 2599 | 138 | 5.3 (4.5, 6.3) |
| McIver (2009) [42] | NSW (Sydney) | | | Female sexual health clinic clients | | Cross-sectional survey | Cervical swab | n.d. | F | ≥18 | 2006–2007 | 175 | 1 | 0.6 (0.1, 3.1)* |
| O’Rourke (2009) [43] | VIC (Melb) | | | Women attending a sexual health clinic | | Clinical audit | Urine/swab | NA | F | 12.2– 80.7  (<25)  (25–34)  (35+) | 2003–2007 | 10,498  4710  4178  1610 | 619  382  192  45 | 5.9 (5.5, 6.4)  8.1 (7.3, 8.9)*  4.6 (4.0, 5.3)*  2.8 (2.0, 3.7)* |
| Franklin (2010) [44] | NSW (Sydney) | | | Sexual health clinic clients | | Clinical audit | Urine/swab | 66‑69 | M/F  F  M | (<25)  (<25)  (<25) | 2004–2008 | 2999  394  235  159 | 281  39  23  16 | 9.4 (8.4, 10.5)*  9.9 (7.1, 13.3)*  9.8 (6.3, 14.3)*  10.1 (5.9, 15.8)* |
| Goller (2010) [45] | VIC (urban and rural) | | | Clients attending family planning or sexual health sentinel sites | | Sentinel surveillance | Any | NA | F  M | 16+ | 2006–2008 | 11351  9280 | 543  678 | 4.8 (4.4, 5.2)*  7.3 (6.8, 7.9)* |
| Guy (2011) [46] | Australia-wide | | | Women and heterosexual men attending sexual health sentinel sites | | Sentinel surveillance | Any | 60 | F  M | n.r. | 2004–2008 | 31190  28530 | 4045  3825 | 13.0 (12.6, 13.3)*  13.4 (13.0, 13.8)* |
| McKechnie (2011)  [47] | NSW (Sydney) | | | Women attending sexual health clinics | | Cross-sectional survey | Urine/swab | 12 | F | 18+  (18–24)  (25–29)  (30–34)  (35+) | 2008–2009 | 216  50  89  36  41 | 8  5  1  2  0 | 3.7 (1.6, 7.2)*  10.0 (3.3, 21.8)*  1.1 (0.0, 6.1)*  5.5 (0.7, 18.7)*  0 (0.0, 8.6)* |
| Vodstrcil (2011) [48] | VIC (Melb) | | | Heterosexual men attending a sexual health clinic | | Clinical audit | Urine/swab | NA | M | All  (<25)  (25–34)  (35+) | 2002–2009 | 17769  4310  7908  5549 | 1310  433  632  245 | 7.37 (6.99, 7.77)  10.1 (9.2, 11.0)  8.0 (7.4, 8.6)*  4.4 (3.9, 5.0)* |
| Walker (2011) [34] | Australia-wide | | | Women attending primary health care clinics | | Cross-sectional survey | Swab | 66 | F | 16–25 | 2007–2008 | 378 | 30 | 7.9 (4.1, 11.8) |
| **Youth Centres** | |  | |  | |  |  |  |  |  |  |  |  |  |
| Rosenthal (2000) [49] | VIC (Melb) | | | Homeless youths accessing a Youth Health Bus or other youth health service | | Cross sectional survey | Tampon; urine | n.r. | F/M | 18–25 | 1999 | 48 | 2 | 4.2 (0.5, 14.3)* |
| Debattista (2002) [18] | QLD (Bris) | | | Disadvantaged youths (non-Indigenous) attending a youth centre | | Clinical audit | Any | NA | F  M | n.r. | 1998–2001 | 47  38 | 5  4 | 10.6 (3.5, 23.1)*  10.5 (2.9, 24.8)* |
| Heal  (2002) [28] | QLD (Mackay) | | | Young people attending youth clinics | | Cross-sectional survey | Urine | 68 | F/M | 18–24 | 2001 | 65 | 8 | 12.3 (5.5, 22.8)* |
| Cole  (2004) [50] | NSW (Sydney) | | | Young people attending a youth health service | | Clinical audit | n.r. | NA | F  M | 14–25 | 2001 | 179  15 | 14  3 | 7.8 (4.3, 12.8)*  20.0 (4.3, 48.1)* |
| Jones  (2004) [51] | VIC (Gee-long) | | | Youths attending a youth health clinic | | Cross-sectional survey | Urine/ swab | 100 | F  M | 12–25 | 2002–2003 | 154  9 | 9  0 | 5.8 (2.7, 10.8)*  0 (0.0, 33.6) |
| Kang  (2006) [52] | NSW (urban and rural) | | | Homeless or at-risk youths attending a youth health service | | Cross-sectional survey | Urine | n.d. | M/F | 14–25 | 2000–2003 | 274 | 16 | 5.8 (3.4, 9.3)** |
| Buhrer-Skinner (2009) [18] | QLD (Towns-ville) | | | Individuals attending a youth health service | | Cross-sectional survey | M: urine  F: urine/ swab | n.r. | F | 16^A^ | 2004–2005 | 23 | 3 | 13 (2.8, 33.6) |
| Gilbert (2009) [53] | NSW (Sydney) | | | Clients attending a youth oriented sexual health service | | Clinical audit | Any | 79–85 | M/F | <24 | 2006–2008  (2006–2007)  (2007–2008) | 305  146  159 | 22  4  18 | 7.2 (4.6, 10.7)*  2.7 (0.8, 6.9)*  11.3 (6.8, 17.3)* |
| Goller (2010) [45] | VIC (urban and rural) | | | Clients attending youth health sentinel sites | | Sentinel surveillance | Any | NA | F  M | 16+ | 2006–2008 | 184  272 | 25  29 | 13.6 (9.0, 19.0)  10.7 (7.3, 15.0) |
| **Other Clinical Settings** | | |  |  | |  |  |  |  |  |  |  |  |  |
| Debattista (2004) [55] | QLD (Bris) | | | Women presenting to a hospital IVF service for investigation of infertility | | Cross-sectional survey | Swab; fallopian tube washings | n.d. | F | n.r. | n.r. | 44 | 1 | 2.3 (0.1, 12.0)* |
| Petersen (2007) [56] | VIC (Melb) | | | Women attending a public colposcopy clinic | | Cross-sectional survey | Swab | 98 | F | 33.7  (≤25)  (>25) | n.r. | 560  137  423 | 12  8  4 | 2.1 (1.1, 3.7)**  5.8 (2.6, 11.2)**  1.0 (0.3, 2.4)** |
| Dykstra (2008) [56] | WA (Perth) | | | People attending a public hospital emergency department, including visitors | | Cross-sectional survey | Urine | n.r. | M/F | 18–25 | n.r. | 823 | 45 | 5.5 (4.0, 7.2)* |
| Goller (2010) [45] | VIC (urban and rural) | | | Clients attending a women’s outpatient clinic | | Sentinel surveillance | Any | NA | F | 16+ | 2006–2008 | 227 | 7 | 3.1 (1.2, 6.3) |
