# Supplementary material for: The prevalence of Chlamydia trachomatis infection in Australia: a systematic review and meta-analysis
Source: BMC Infect Dis. 2012 May 14;12:113. doi: 10.1186/1471-2334-12-113 (PMC3462140; doi:10.1186/1471-2334-12-113)
Supplement: Additional file 2: — Studies reporting chlamydia prevalence data, identified in pregnant women. Studies are presented in order of publication year and author. * Confidence intervals calculated by authors. ** Re-calculated confidence intervals differ from those reported. A Median. Melb, Melbourne; NA, not applicable; n.d., not determined; n.r., not reported; NSW, New South Wales; QLD, Queensland; VIC, Victoria; WA, Western Australia. Participant numbers reflect numbers from which epidemiological data was calculated, with sub-group numbers (e.g. by age or year) in brackets. (DOC 41 kb) [file 1471-2334-12-113-S2.doc]

## Additional File 2 - Studies reporting chlamydia prevalence data, identified in pregnant women

Studies are presented in order of publication year and author. ***** Confidence intervals calculated by authors. ** Re-calculated confidence intervals differ from those reported. ^A^ Median. Melb, Melbourne; NA, not applicable; n.d., not determined; n.r., not reported; NSW, New South Wales; QLD, Queensland; VIC, Victoria; WA, Western Australia. Participant numbers reflect numbers from which epidemiological data was calculated, with sub-group numbers (e.g. by age or year) in brackets.

| **Study** | **Location** | **Participants** | **Study design** | **Specimen type** | **Response rate (%)** | **Age (years)** | **Study period** | **Tested (n)** | **Positive (n)** | **Prevalence**  **% (95% CI)** |
| --- | --- | --- | --- | --- | --- | --- | --- | --- | --- | --- |
| Garland (2000) [58] | VIC (Melb) | Women attending a hospital for legal termination of pregnancy | Cross-sectional survey | Urine/ tampon/ swab | 99.8 | n.r. | 1996–1997 | 1175 | 33 | 2.8 (1.9, 3.9)* |
| Garrow (2002) [59] | WA (Kimberly, remote) | Women attending gynaecological investigation in remote communities | Cross-sectional study | Urine/ swab | n.d. | 28.8 | 2000–2001 | 70 | 2 | 2.9 (0.4, 9.9)** |
| Quinlivan (2002) [60] | Australia (urban) | Pregnant teenagers attending a hospital antenatal clinic | Prospective cohort study | Swab | 91 | 12–17 | 1998–2000 | 456 | 42 | 9.2 (6.7, 12.2)* |
| Quinlivan (2004) [61] | Australia (urban) | Pregnant teenagers attending hospital antenatal services | Prospective cohort study | Swab | 92 | <18 | n.r. | 457 | 26 | 5.7 (3.8, 8.2)* |
| Cheney (2006) [62] | NSW (Sydney) | Women attending a hospital antenatal service | Cross-sectional survey | Urine | 96 | ≥16  (16–24) | 2004 | 239  64 | 8  6 | 3.4 (1.5, 6.5)**  9.4 (3.5, 19.3)* |
| Panaretto (2006) [63] | QLD (Towns-ville) | Pregnant, urban Indigenous women attending community health services | Cross-sectional survey | Urine/ tampon | 88 | All  (<25) | 2000–2006 | 403  223 | 58  49 | 14.4 (11.1, 18.2)**  22.0 (16.7, 28.0)* |
| Lenton  (2007) [64] | NSW (rural, remote) | Women attending antenatal services | Cross-sectional survey | Urine | 52 | 16–46  (<25) | 2004–2006 | 218 | 6 | 2.7 (1.0, 5.9)  4.5 (1.5, 10.3) |
| Cheney (2008) [65] | NSW (Sydney) | Women attending a hospital antenatal service | Clinical audit | Urine | 58 | ≤20 | 2003–2006 | 212 | 29 | 13.7 (9.4, 19.1)* |
| Combs (2008) [66] | WA (Perth) | Women attending hospital antenatal services | Clinical audit | Any | NA | n.r. | 2007 | 254 | 10 | 3.9 (1.5, 6.3) |
| Chen  (2009) [67] | VIC (Melb) | Pregnant women attending antenatal services | Cross-sectional survey | Urine | 88 | 16–25 | 2006–2007 | 987 | 32 | 3.2 (1.8, 5.9) |
| Ward (2010) [68] | Australia-wide | Indigenous clients attending an antenatal service | Sentinel surveillance | Any | 88.3 | All | 2009 | 447 | 55 | 12.3 (9.4, 15.7)* |
