# Supplementary material for: The prevalence of Chlamydia trachomatis infection in Australia: a systematic review and meta-analysis
Source: BMC Infect Dis. 2012 May 14;12:113. doi: 10.1186/1471-2334-12-113 (PMC3462140; doi:10.1186/1471-2334-12-113)
Supplement: Additional file 3: — Studies reporting chlamydia prevalence data, identified in Indigenous Australians. Studies are presented in order of publication year and author. * Confidence intervals calculated by authors. ** Re-calculated confidence intervals differ from those reported. A Median. Bris, Brisbane; F, female; GP, general practice, general practitioner; M, male; Melb, Melbourne; NA, not applicable; n.d., not determined; n.r., not reported; NSW, New South Wales; NT, Northern Territory; QLD, Queensland; SA, South Australia; WA, Western Australia. Participant numbers reflect numbers from which epidemiological data was calculated, with sub-group numbers (e.g. by age or year) in brackets. (DOC 53 kb) [file 1471-2334-12-113-S3.doc]

## Additional File 3 - Studies reporting chlamydia prevalence data, identified in Indigenous Australians

Studies are presented in order of publication year and author. ***** Confidence intervals calculated by authors. ** Re-calculated confidence intervals differ from those reported. ^A^ Median. Bris, Brisbane; F, female; GP, general practice, general practitioner; M, male; Melb, Melbourne; NA, not applicable; n.d., not determined; n.r., not reported; NSW, New South Wales; NT, Northern Territory; QLD, Queensland; SA, South Australia; WA, Western Australia. Participant numbers reflect numbers from which epidemiological data was calculated, with sub-group numbers (e.g. by age or year) in brackets.

| **Study** | **Location** | **Participants** | **Study design** | **Specimen type** | **Response rate (%)** | **Sex** | **Age (years)** | **Study period** | **Tested  (n)** | **Positive (n)** | **Prevalence**  **% (95% CI)** |
| --- | --- | --- | --- | --- | --- | --- | --- | --- | --- | --- | --- |
| Miller (1999) [69]; Huang (2008) [70] | SA (remote Central Australia) | Indigenous men and women | Serial cross-sectional survey | Urine | n.d. | F  M | 12–40 | 1997–2006  (1997–1998)  (1997)  (1998)  1997–2006  (1997–1998)  (1997)  (1998) | 1019  546  473  874  442  443 | 75  41  34  65  34  31 | 7.4 (5.8, 9.1)*  7.5 (5.5, 10.1)*  7.2 (5.1, 10.0)*  7.4 (5.8, 9.4)*  7.7 (4.5, 10.7)*  7.2 (5.0, 10.2)* |
| Debattista (2002) [18] | QLD (Bris) | Indigenous youths detached from formal education | Clinical audit | Urine | 30–50 | F  M | 15–18 | 1998–2001 | 154  110 | 33  13 | 21.4 (15.2, 28.7)*  11.8 (6.4, 19.4)* |
| Garrow (2002) [59] | WA (Kimberly, remote) | Remote women undergoing gynaecological exams | Cross- sectional survey | Urine/ swab | n.d. | F | 28.8 | 2000–2001 | 303 | 28 | 9.2 (6.2, 13.1)* |
| Knox  (2002) [71] | Central Australia (urban/remote) | Indigenous women from urban and remote areas of central Australia | Cross-sectional survey | Urine/ swab/ tampon | n.r. | F | n.r. | 1998–1999 | 313 | 36 | 11.5 (8.3, 15.7) |
| Miller (2003) [72] | QLD (regional/ remote) | Indigenous people attending Indigenous health services | Cross-sectional survey | Urine | n.d. | F  M | ≥15  (15–19)  (20–24)  (25–29)  (30–34)  (35–39)  (40+)  ≥15  (15–19)  (20–24)  (25–29)  (30–34)  (35–39)  (40+) | 1998–2000 | 1456  169  181  187  186  162  571  1361  149  183  179  164  157  529 | 158  58  40  17  12  12  19  117  28  34  21  12  8  14 | 10.9 (7.1, 12.1)*  34.3 (27.2, 42.0)*  22.1 (16.3, 28.9)*  9.1 (5.4, 14.2)*  6.5 (3.4, 11.0)*  7.4 (3.9, 12.6)*  3.3 (2.0, 5.1)*  8.6 (7.1, 10.1)*  18.8 (12.9, 26.0)*  18.6 (13.2, 25.0)*  11.7 (7.4, 17.4)*  7.3 (3.8, 12.4)*  5.1 (2.2, 9.8)*  2.6 (1.5, 4.4)* |
| Latif  (2004) [73] | Central Australia | Individuals in 27 Indigenous communities in central Australia | Cross-sectional survey | F: swab; M: urine | n.d. | F  M | 13–67  13–54 | 2004 | 694  525 | 71  46 | 10.2 (8.1, 12.7)*  8.8 (6.5, 11.5)* |
| Panaretto (2006) [63] | QLD (Townsville) | Pregnant, urban Indigenous women attending community health services | Cross-sectional survey | Urine/ tampon | 88 | F | All  (<20)  (20–24)  (25–34)  (35+) | 2000–2003 | 403  98  125  101  79 | 58  31  18  8  1 | 14.4 (11.1, 18.2)**  32.6 (23.3, 41.9)  14.4 (8.2, 20.6)  7.9 (2.6, 13.2)  1.7 (1.2, 4.6) |
| Panaretto (2006) [74] | QLD (Townsville) | Urban Indigenous and non-Indigenous women attending community health services | Cross-sectional survey | Tampon | 36 | F | 20–69 | 2002–2004 | 145 | 7 | 4.8 (2.0, 9.7)* |
| Lenton (2007) [64] | NSW (rural, remote) | Women attending an antenatal check-up | Cross-sectional survey | Urine | 52 | F | 18–30 | 2004–2006 | 44 | 4 | 9.1 (2.5, 21.7) |
| Buhrer-Skinner (2009) [23] | QLD (Townsville) | Clients of outreach screening services | Cross-sectional survey | M: urine  F: urine/ swab | 24 | M/F | 15^A^ | 2004–2005 | 20 | 3 | 15 (3.2, 37.9) |
| Spurling (2009) [75] | QLD (Brisb) | Clients attending an Indigenous health service | Cross-sectional survey | n.r. | 81 | F/M | n.r. | 2007–2008 | 413 | 18 | 4.4 (2.6, 6.8)* |
| Fairbairn (2010) [76] | NT (Alice Springs) | Indigenous women attending an emergency department | Cross-sectional survey | Urine, swab | 84 | F | 16–35 | 2007 | 213 | 19 | 8.9 (5.5, 13.6)* |
| Franklin (2010) [44] | NSW (Sydney) | Sexual health clinic clients | Clinical audit | Urine/ swab | 51 | M/F | NA | 2004–2008 | 60 | 4 | 6.7 (1.8, 16.2)* |
| Ward (2010) [68]; Goller (2010) [77] | Australia-wide | Indigenous clients of surveillance sites  (General practice clinics)  (Sexual health centres)  (Antenatal clinics)  (Aboriginal community controlled health services) | Sentinel surveil-lance | Any | 3.95  62.8  88.3  17.7 | F/M  F/M  F/M  F  F/M | Any | 2009 | 863  904  447  1337 | 63  134  55  87 | 7.3 (5.7, 9.2)*  14.8 (12.6, 17.3)*  12.3 (9.4, 15.7)*  6.5 (5.2, 8.0)* |
| Templeton (2010 ) [78] | NSW (Dubbo) | Detainees of a juvenile detention centre (87% Indigenous) | Clinical audit | Urine | n.r. | M | 14–20 | 2000–2004 | 86 | 14 | 16.3 (9.2, 25.8)** |
