# Supplementary material for: The prevalence of Chlamydia trachomatis infection in Australia: a systematic review and meta-analysis
Source: BMC Infect Dis. 2012 May 14;12:113. doi: 10.1186/1471-2334-12-113 (PMC3462140; doi:10.1186/1471-2334-12-113)
Supplement: Additional file 4: — Studies reporting chlamydia prevalence data, identified in men who have sex with men. Studies are presented in order of publication year and author. * Confidence intervals calculated by report authors. ** Re-calculated confidence intervals differ from those reported. A Median. ACT, Australian Capital Territory; HIV+, HIV-positive; HIV–, HIV-negative; Melb, Melbourne; NA, not applicable; n.d., not determined; n.r., not reported; NSW, New South Wales; Ph, Pharynx; QLD, Queensland; R, rectum; SHC, sexual health centre; U, urethra; VIC, Victoria. Participant numbers reflect numbers from which epidemiological data was calculated, with sub-group numbers (e.g. by age or year) in brackets. (DOC 54 kb) [file 1471-2334-12-113-S4.doc]

## Additional File 4 - Studies reporting chlamydia prevalence data, identified in men who have sex with men

Studies are presented in order of publication year and author. ***** Confidence intervals calculated by report authors. ** Re-calculated confidence intervals differ from those reported. ^A^ Median. ACT, Australian Capital Territory; HIV+, HIV-positive; HIV–, HIV-negative; Melb, Melbourne; NA, not applicable; n.d., not determined; n.r., not reported; NSW, New South Wales; Ph, Pharynx; QLD, Queensland; R, rectum; SHC, sexual health centre; U, urethra; VIC, Victoria. Participant numbers reflect numbers from which epidemiological data was calculated, with sub-group numbers (e.g. by age or year) in brackets.

| **Study** | **Location** | **Participants** | **Study design** | **Specimen type** | **Response rate (%)** | **Age  (years)** | **Study period** | **Tested  (n)** | **Positive (n)** | **Prevalence**  **% (95% CI)** |
| --- | --- | --- | --- | --- | --- | --- | --- | --- | --- | --- |
| Debattista (2000) [79] | QLD (Bris) | MSM attending sex-on-premises venues and a sexual health centre | Cross-sectional survey | U/Ph  U: urine  Ph: swab | ~50 | n.r. | 1999 | 90  90  90 | 4  3  1 | 4.4 (1.2, 11.0)*  3.3 (0.7, 9.4)**  1.1 (0.3, 6.0)** |
| Debattista (2002) [80] | QLD (Bris) | MSM attending inner-city homosexual entertainment venues | Cross-sectional survey | U/Ph  U: urine  Ph: swab | ~40 | n.r. | 2000 | 184  161 | 8  0 | 4.3 (1.9, 7.8)  0 (0, 2.3) |
| Lister (2003) [81] | VIC (Melb) | MSM attending men-only saunas | Cross-sectional survey | U/R/Ph  U: urine  R: swab  Ph: swab | 24 | All  (<30)  (30–39)  (40–49)  (50+) | 2001–2002 | 521  105  166  132  118  511  507  521 | 39  10  17  6  6  9  30  3 | 7.5 (5.4, 10.1)*  9.5 (4.7, 16.8)*  10.2 (6.1, 16.9)*  4.6 (1.7, 9.6)*  5.1 (1.9, 10.7)*  1.8 (0.8, 3.3)  5.9 (4, 8)  0.6 (0.1, 1.7) |
| Lister (2004) [82] | VIC (Melb) | MSM attending a sexual health clinic | Clinical audit | U/R/Ph  U: urine  R: swab  Ph: swab | NA | NA | 2001–2002 | 80  34  30 | 4  5  0 | 5.0 (1.4, 12.3)*  14.7 (5.0, 31.1)*  0.0 (0.0, 11.6)* |
| Hocking (2005) & (2006) [38] [39] | VIC (Melb) | Sexual health clinic clients | Clinical audit | U/R: urine/swab | NA | All  (<20)  (20–24)  (25–29)  (30–34)  (35–39)  (40+) | 2002–2003 | 614  28  133  145  108  80  120 | 56  3  10  13  15  10  5 | 9.1 (7.0, 11.7)  10.7 (2.3, 28.2)  7.5 (3.7, 13.4)  9.0 (4.9, 14.8)  13.9 (8.0, 21.9)  12.5 (6.2, 21.8)  4.2 (1.4, 9.5) |
| Lister (2005) [83] | VIC (Melb) | Males attending men-only saunas | Clinical outreach | U/R/Ph  U: urine  R: swab  Ph: swab | 86–89 | Mean 30–41 | 2001–2002 | 138  142  144 | 2  11  3 | 1.5 (0.2, 5.1)**  7.8 (3.9, 13.4)**  2.1 (0.4, 6.0)** |
| Lister (2005) [84] | VIC (Melb) | MSM attending a sexual health clinic | Clinical audit | U/R/Ph  U: urine  R: swab  Ph: swab | 77 | NA | 2001–2003 | 1160  804  484 | 24  39  2 | 2.1 (1.3, 3.1)*  4.9 (3.5, 6.6)*  0.4 (0.1, 1.5)* |
| Currie (2006) [85] | ACT (Can-berra) | MSM attending a sexual health clinic and a general practice with a high MSM caseload | Clinical audit | U/R/Ph  U: urine  R: swab  Ph: swab | NA | 34.8^A^  (14–74) | 2001–2004 | 157  153  130  151 | 16  5  12  4 | 10.2 (5.9, 16.0)  3.3 (1.1, 7.5)  9.2 (4.2, 15.6)  2.6 (0.7, 6.6) |
| Hamlyn (2006) [86] | NSW (Sydney) | MSM receiving emergency HIV prophylaxis at a hospital | Cross-sectional survey | U/R/Ph  U: urine  R: swab  Ph: swab | 85 | 33  (18–62) | 2001–2004 | 253  200  244  200 | 2  11  1 | 1.0 (0.1, 3.6)*  4.5 (2.3, 7.9)*  0.5 (0.1, 2.8)* |
| Jin  (2007) [87]; Templeton (2008) [88] | NSW (Sydney) | MSM (HIV-positive and HIV-negative) recruited from the community | Cohort study | U/R/Ph  U: swab  R: swab  Ph: swab  U/R  U: swab  R: swab | 79-88% | n.r. | 2001–2006 | HIV–  1192  1186  1227  HIV+  225  222 | 11  52  13    5  13 | 0.9 (0.5, 1.6)  4.4 (3.3, 5.7)*  1.1 (0.6, 1.8)  2.2 (0.7, 5.1)  5.9 (3.1, 9.8) |
| McNulty (2008) [89] | NSW (Sydney) | Contacts of chlamydia, non-gonococcal urethritis and PID attending a sexual health centre | Clinical audit | Any | NA | n.r. | 2003–2006 | 188 | 54 | 28.7 (22.4, 35.8)* |
| Teague (2008) [90] | VIC (Melb) | HIV-positive MSM attending HIV clinics based at a sexual health clinic and a hospital | Clinical audit | U/R/Ph  U: urine/swab  R: swab  Ph: swab | NA | 47  (25–81) | 2006 | 505  505  505 | 9  20  0 | 1.8 (0.8, 3.4)*  4.0 (2.4, 6.1)*  0 (0, 0.7)* |
| Franklin (2010) [44] | NSW (Sydney) | Sexual health clinic clients | Clinical audit | U/R: urine/swabs | 78 | NA  (20–24) | 2004–2008 | 1015 | 83 | 8.2 (6.6, 10.1)  12.4 |
| Goller (2010)  [45] | VIC (urban and rural) | MSM attending sexual health and gay men’s health sentinel sites | Sentinel surveillance | Any | NA | 16+ | 2006–2008 | 9202 | 528 | 5.7 (5.3, 6.2) |
| Guy (2011) [46];  Guy (2011)  [91] | Australia-wide (urban and rural) | MSM attending sexual health sentinel sites | Sentinel surveillance | U/R/Ph  Any  U: urine/swab  R: swab | NA | n.r. | 2004–2008 | All clinics:  10207  Sydney SHS:  4099  4043  3650 | 1186  373  174  245 | 11.6 (11.0, 12.3)*  9.1 (8.2, 10.0)*  4.3 (3.7, 5.0)  6.7 (5.9, 7.5) |
| Vodstrcil (2011) [48] | VIC (Melb) | Homosexual men attending a sexual health clinic | Clinical audit | U/R  U: urine/swab  R: swab | NA | 31^A^  (15–84) | 2002–2009 | 8328  7977  6237 | 295  334 | 3.7 (3.3, 4.1)  5.4 (4.8, 6.0) |
