# Supplementary material for: The prevalence of Chlamydia trachomatis infection in Australia: a systematic review and meta-analysis
Source: BMC Infect Dis. 2012 May 14;12:113. doi: 10.1186/1471-2334-12-113 (PMC3462140; doi:10.1186/1471-2334-12-113)
Supplement: Additional file 5: — Studies reporting chlamydia prevalence data, identified in high-risk populations. Studies are presented in order of publication year and author. * Confidence intervals calculated by authors. ** Re-calculated confidence intervals differ from those reported. A Median. Bris, Brisbane; broth, brothel; CBD, central business district; F, female; M, male; Melb, Melbourne; NA, not applicable; n.d., not determined; n.r., not reported; NSW, New South Wales; QLD, Queensland; st, street; WA, Western Australia. Participant numbers reflect numbers from which epidemiological data was calculated, with sub-group numbers (e.g. by age or year) in brackets. (DOC 48 kb) [file 1471-2334-12-113-S5.doc]

## Additional File 5 - Studies reporting chlamydia prevalence data, identified in high-risk populations

Studies are presented in order of publication year and author. ***** Confidence intervals calculated by authors. ** Re-calculated confidence intervals differ from those reported. ^A^ Median. Bris, Brisbane; broth, brothel; CBD, central business district; F, female; M, male; Melb, Melbourne; NA, not applicable; n.d., not determined; n.r., not reported; NSW, New South Wales; QLD, Queensland; st, street; WA, Western Australia. Participant numbers reflect numbers from which epidemiological data was calculated, with sub-group numbers (e.g. by age or year) in brackets.

| **Study** | **Location** | | | **Participants** | **Study design** | **Specimen type** | **Response rate (%)** | **Sex** | **Age**  **(years)** | **Study period** | **Tested  (n)** | **Positive (n)** | **Prevalence**  **% (95% CI)** |
| --- | --- | --- | --- | --- | --- | --- | --- | --- | --- | --- | --- | --- | --- |
| **Sex Workers** | |  | |  |  |  |  |  |  |  |  |  |  |
| Morton (1999) [92] | VIC (Melb) | | | Sex workers attending a community outreach program | Cross-sectional survey | F: tampon; M: urine | 78 | M/F | 26^A^  (17– 41) | n.r. | 63 st  753 broth | 1  9 | 1.6 (0.43, 8.5)  1.2 (0.54, 2.25) |
| Morton (2002) [93] | VIC (Melb) | | | Sex workers attending youth or sexual health clinics | Cross-sectional survey | F: tampon; M: urine | n.d. | M/F | 16-43  n.r. | n.r. | 102 st  1664 broth | 7  44 | 6.86 (2.8, 13.6)  2.64 (1.92, 3.53) |
| Hocking (2005) [38] | VIC (Melb) | | | Sexual health clinic clients | Clinical audit | Urine/ swab | NA | M/F | n.r. | 2002–2003 | 302 | 10 | 3.3 (1.6, 6.0) |
| O’Connor (2008) [94] | WA (Perth) | | | Sex workers working at illegal brothels | Cross-sectional survey | Tampon | 87 | F | n.r. | 2007 | 175 | 5 | 2.9 (0.9, 6.5)* |
| Franklin (2010) [44] | NSW (Sydney) | | | Sexual health clinic clients | Clinical audit | Urine/ swab | 83 | M/F  F  M | NA | 2004–2008 | 193 | 16 | 8.3 (4.8, 13.1)  7.8  11.1 |
| **Prisoners** |  | | |  |  |  |  |  |  |  |  |  |  |
| Debattista (2002) [18] | QLD (Bris) | | | Youths at an adolescent detention centre | Clinical audit | Any | NA | F  M | n.r | 1998–2001 | 48  163 | 11  23 | 22.9 (12.0, 37.3)*  14.1 (9.2, 20.4)* |
| Mak  (2004) [95] | WA (Kimberley, remote) | | | Men screened on admission to prison | Cross-sectional survey | n.r. | n.d. | M | n.r. | 1998–1999 | 93 | 3 | 3 (1, 9) |
| Watkins (2009) [96] | WA (urban and regional) | | | Prisoners | Clinical audit | n.r. | 49 | F/M  (F)  (M) | 12–57  Juvenile  Juvenile | 2005–2007 | 466 | 34 | 7.3 (4.9, 9.7)  20.2 (12.7, 30.3)*  2.0 (0.4, 7.7)* |
| Templeton (2010 ) [78] | NSW (Dubbo) | | | Detainees of a juvenile detention centre (87% Indigenous) | Clinical audit | Urine | n.r. | M | 14–20 | 2000–2004 | 86 | 14 | 16.3 (9.2, 25.8)** |
| **Other High-risk** | | |  |  |  |  |  |  |  |  |  |  |  |
| Quinlivan (2002) [60] | Australia (urban) | | | Pregnant teenagers attending a hospital antenatal clinic  (Multi-drug user)  (Marajuana-user)  (No drugs) | Prospective cohort study | Swab | 91 | F | 12–17 | 1998–2000 | 456  31  62  363 | 42  8  6  28 | 9.2 (6.7, 12.2)*  25.8 (11.9, 44.6)*  9.7 (3.6, 19.9)*  7.7 (5.2, 11.0)* |
| Bradshaw (2005) [97] | VIC (Melb CBD) | | | Street-based injecting drug users | Cross-sectional survey | Urine/ swab | 68 | M/F | 24.2^A^  (17–45) | 1999–2002 | 213 | 13 | 6 (3, 10) |
| McNulty (2008) [89] | NSW (Sydney) | | | Contacts of chlamydia, non-gonococcal urethritis and PID attending a sexual health centre | Clinical audit | Any | NA | M/F | n.r. | 2003–2006 | 438 | 145 | 33.1 (28.7, 37.7)* |
